# Biological and Physical Characterization of Surface-Modified Grade V Titanium Alloy

**DOI:** 10.1155/2024/6662866

**Published:** 2024-08-21

**Authors:** Mahesh Kakunje, Supriya Nambiar, Arun M. Isloor, Shamaprasada Kabekkodu, Udaya Bhat

**Affiliations:** ^1^ Department of Orthodontics and Dentofacial Orthopedics Manipal College of Dental Sciences Mangalore Manipal Academy of Higher Education, Manipal 576104, Karnataka, India; ^2^ Membrane and Separation Technology Laboratory Department of Chemistry National Institute of Technology Karnataka, Surathkal, Mangalore 575 025, India; ^3^ Department of Biotechnology School of Life Sciences Manipal Academy of Higher Education, Manipal 576104, Karnataka, India; ^4^ Department of Metallurgical and Materials Engineering National Institute of Technology Karnataka, Surathkal, Mangalore 575 025, India

## Abstract

Surface modification and biomimetic approaches have been widely used to enhance bioinert substances. It is not very clear whether surface alterations and polymer coatings on titanium make it more biologically active and enhance cell adhesion. We tried to focus on the physical and biological characterization of surface-modified titanium disks. Four different surface modifications were done for the titanium disks, ranging from acid etching, sandblasting, polydopamine coating, and polydopamine-based chitosan coating, and were compared with disks without any surface modification. The disks were studied for physical characteristics like surface roughness and contact angle. Human gingival fibroblasts were used to investigate the biological effects of surface modification of titanium alloy surfaces. The wettability of chitosan-coated, acid-etched, and polydopamine-coated titanium was much better than that of the sandblasted surface, indicating that surface energy was higher for acid-etched and coated surfaces than others. The cell seeding with fibroblasts showed increased adhesion to the smoother surfaces as compared to the rougher surfaces. Polydopamine coatings on titanium disks showed the most favorable physical and biological properties compared to others and can be a good surface coating for in vivo implants.

## 1. Introduction

Titanium is considered the most ideal material for dental implants. However, titanium surfaces if surface-modified can add to the biological response, biocompatibility, and surface properties. The nanometer roughness scale on implant materials will help to provide better integration with bone. Surface modifications that are currently done are surface hydroxyapatite-coating, acid etching, sandblasting, porous sintering, oxidization, and plasma-spraying. Bioactive coatings like calcium phosphate, bioactive glass, enzymes, and proteins help develop strong tissue attachments [1]. Bioactive polymers like chitosan (deacetylated derivative of chitin) and polymerized dopamine (polydopamine, PDA) have recently gained popularity. Chitosan is a positively charged polysaccharide from the chitin family which is biocompatible and biodegradable; its degradation products are nontoxic and nonimmunogenic. It is bioadhesive and bacteriostatic and acts as a chelating agent, antioxidant, and hemostatic agent [2]. Chitosan can control bleeding by combining with procoagulants like hyaluronic acid and tranexamic acid [3, 4]. Polydopamine is a biopolymer synthesized to mimic dopamine found in the human body. Therefore, it exhibits excellent biocompatibility and possesses tissue engineering characteristics, allowing it to be easily coated on various surfaces, making it widely applicable in the medical field [5]. Polydopamine prepared as a layer of polymerized dopamine in a weak alkaline solution has been used as a versatile biomimetic surface modifier and an immobilizing macromolecule. Titanium is widely used in medicine and dentistry for biomedical devices, and there has been considerable research on improving the surface characteristics of titanium to minimize untoward effects. This is evident in the dental field as cases of peri-implantitis due to biofilm formation leading to failure of the titanium implants. Alterations and improvements of the titanium surfaces could enhance the biological responses of the soft tissue components surrounding it. Therefore, this study aimed to assess the surface and biological characteristics like surface morphology, roughness, wettability, and cell adhesion properties of surface-modified titanium.

## 2. Materials and Methods

**Figure 1:**
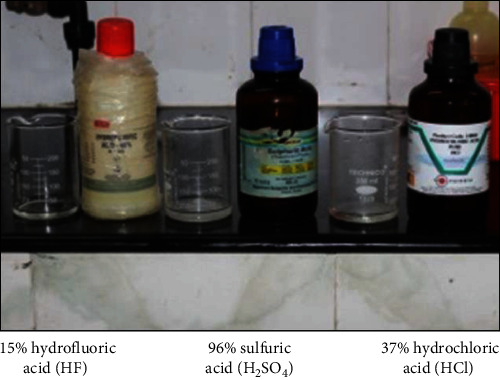
The acids used for etching of titanium disks.

**Figure 2:**
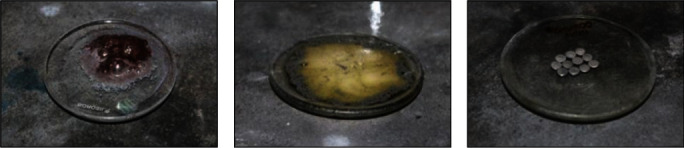
Acid-etched titanium disk.

The Institutional Ethics Committee cleared this research (IRB protocol no. 15124), and the sample comprised 25 titanium alloy disks (Ti-6Al-4V) (SK Surgicals, Pune, India) divided into five groups based on the surface modifications done—the first group (A) comprised of five plain titanium disks with no surface treatment. The second group (B) had five titanium disks which were cleaned thoroughly twice to remove any residual alumina on the surface after which acid etching was done using 15% hydrofluoric acid (HF) (Nice Chemicals, Cochin), 96% sulfuric acid (H_2_SO_4_) (Spectrum Reagents and Chemicals, Cochin), and 37% hydrochloric acid (HCl) (Spectrum Reagents and Chemicals, Cochin (Figure 1) and deionizing solution of 20% sodium bicarbonate in glass plates. All five samples were placed in the glass plates, and the time stopper was set for 2 min. Hydrofluoric acid was slowly poured over disks, and at the end of 5 s, the disks were removed from the glass plate and transferred to another glass plate. A bicarbonate solution was poured over the disks for deionization of residual acids. The samples were kept for 5 min for deionization. Later, the samples were subjected to the next sequence of acid, sulfuric acid for 6 min, and deionized with bicarbonate solution for 5 min. A third sequence of acid etching with hydrochloric acid for 6 min following deionization was done. Finally, it was dried in a furnace set at 100°C for 1 min. (Figure 2). The third group comprised of sandblasted titanium disks. The disks were attached to the molding wax and sandblasted with 110 *μ*m alumina for 4 min at a 4 kg/m^2^ pressure. The sandblasted samples were cleaned with ultrasonic cleaner to remove the residue over the surface and then stored in an isotonic saline solution. The fourth group comprised five titanium disks coated with chitosan over polydopamine. The titanium alloy samples were acid etched and cleaned by ultrasonic cleaner to remove the residues. Later the samples were dipped in an aqueous dopamine solution (1 mg/mL) overnight in a darkroom at 20°C. It was then rinsed with distilled water to remove free dopamine and dried with an air dryer. The dopamine-coated titanium alloys were then subjected to a 3% glutaraldehyde aqueous solution overnight at 20°C and later rinsed with distilled water to remove unbounded glutaraldehyde. The above titanium alloys were then immersed in 1.5 mg/mL of chitosan in 0.1 M acetic acid solution to facilitate bonding between the aldehyde groups on the titanium alloy surface and the amino groups of chitosan molecules and later air dried.

The fifth group comprised five titanium disks coated with polydopamine. The titanium alloy samples were acid etched and cleaned with ultrasonic cleaner to remove the residue. They were dipped in 1 mg/mL of aqueous dopamine solution overnight in a darkroom at 20°C, rinsed with distilled H_2_O to remove free dopamine, and then air dried.

**Figure 3:**
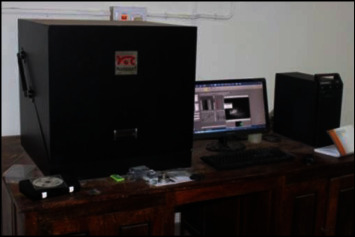
Atomic force microscopy machine.

**Figure 4:**
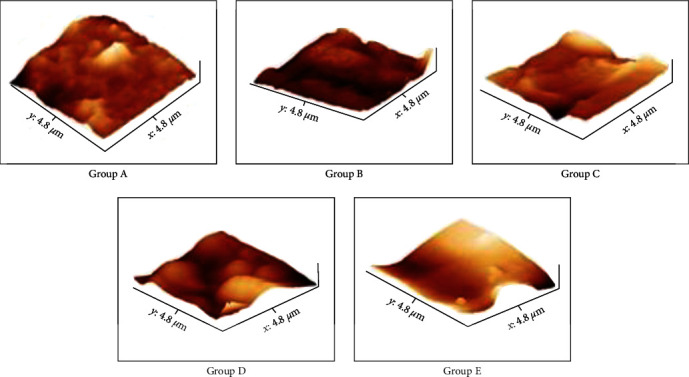
AFM images of roughness of the titanium disk for each groups.

**Figure 5:**
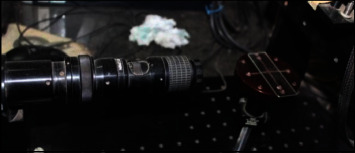
Goniometer for contact angle measurement.

**Figure 6:**
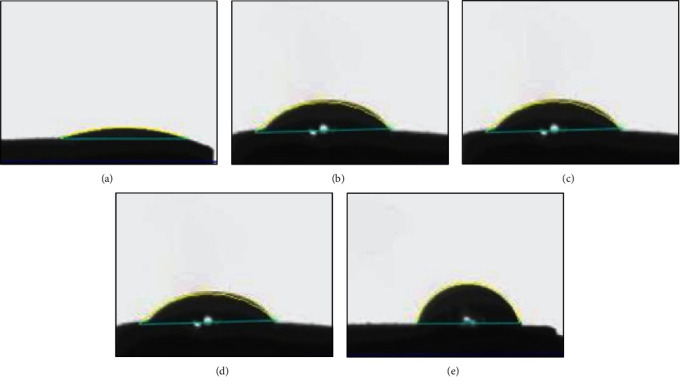
CCD digitized images of the contact angles as measured by the goniometer for the titanium disk of each groups. (a) Plain titanium disk. (b) Acid-etched titanium disk. (c) Sandblasted titanium disk. (d) Chitosan-coated titanium disk. (e) Polydopamine-coated titanium disk.

The disks from each group were subjected to quantitative evaluation using atomic force microscopy at Mangalore University, Konaje, India (Figure 3). The root mean square (RMS) roughness (Rq) was measured using the mean peak height of the surfaces, and the total roughness height at 4.8 *µ*m (Figure 4). The samples were cleaned ultrasonically with acetone/ethanol as the medium. The sample was placed on the sample stage, and the illumination lamp and CCD camera were turned on. After the fixed volume pipette was stabilized, 5 *µ*L of distilled water was dropped over the sample. The contact angles were measured using a goniometer (Figures 5 and 6) automated with image analysis software dpiMax (DataPhysics Instrument, USA). Eight timed measurements over a 15-s interval were made for each surface of surface type, and all the analyses were performed at the temperature of 25°C and 86% humidity.

**Figure 7:**
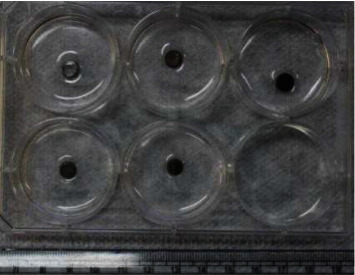
Culture plates of titanium disks after cell seeding.

**Figure 8:**
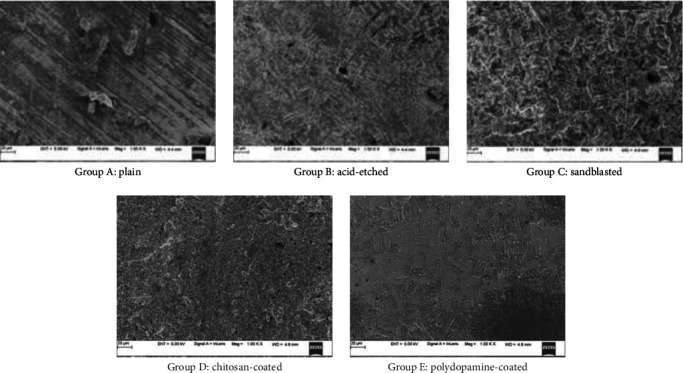
SEM of surface-modified titanium.

**Figure 9:**
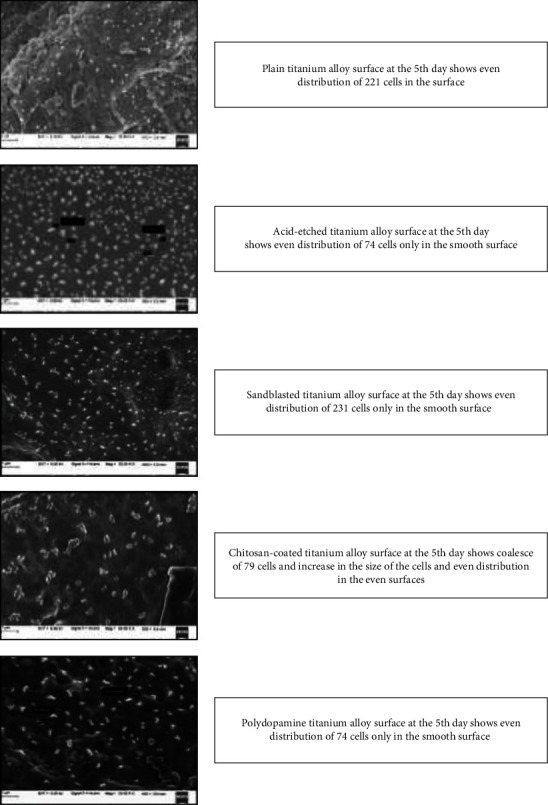
After 120 hr (5th day) of fibroblast seeding, scanning electron microscopy observation at 1 mm.

Human gingival fibroblasts (School of Life Sciences, Manipal) were used in vitro to investigate the effects of titanium alloy surfaces and their modified surfaces on soft tissue response. Fibroblasts were cultured on titanium alloy disks of all five groups at two time periods of 24 and 120 hr. The fibroblasts were cultured in DMEM (Himedia, India) containing 10% FBS. The cells were subcultured according to standard laboratory procedures. The disks were sterilized using UV inside the biosafety cabinet hood. For each experiment, 5,000 cells were seeded on the surface of the disk inside 6-well cell culture plates for indicated times in the presence of DMEM containing 10% FBS (Figure 7). Cells were rinsed with phosphate-buffered saline and were fixed with 3% paraformaldehyde for 10 min followed by extensive rinsing with phosphate-buffered saline. Fibroblasts on polished titanium disks were used as controls. Photographs of disks with different surfaces were obtained after incubation with the fibroblasts. (Figures 8 and 9). Radioimmunoprecipitation assay (RIPA) buffer containing protease inhibitor cocktail (Calbiochem, USA) was used for protein extraction. The concentration of the total protein was estimated by Bradford assay kit (Sigma–Aldrich, USA). The principle of this assay is that the binding of the protein to Coomassie dye brilliant blue G-250 under acidic conditions results in a color change from brown to blue.

The disks containing the cells were incubated with RIPA buffer for 30 min at room temperature. Following this, the supernatant was transferred to a fresh 1.5 mL microcentrifuge tube (Tarson, India) and centrifuged at 12,000 rpm for 15 min and stored at 4°C. The protein assessment was done for day 1 and day 5. For the protein assessment assay on day 1, the supernatant was transferred to a fresh tube, and protein concentration was measured using a visible light spectrophotometer for the absorbance at 595 nm in a Varioskan multimode reader (Thermo-Fisher Scientific, USA), and the same was repeated for the supernatant stored at 4°C. The data was imported to Microsoft Excel (Microsoft, USA) for subsequent analysis. Descriptive statistics including mean, standard deviation, and *p* value were calculated for contact angle and surface roughness. A *p* value less than 0.001 was obtained for both the parameters which was considered significant. The values obtained from the atomic force microscopy and goniometer were analyzed using a one-way ANOVA test. The values obtained from the ANOVA test and post hoc Tukey test were used for the comparison of the roughness and contact angle (Tables 1 and 2). Paired *t*-test was done for the protein level assessment. All the analysis was done in SPSS Software version 20.0

## 3. Results and Observations

The contact angle between the five groups showed that the mean values of sandblasted disks were the highest followed by polydopamine-coated, chitosan-coated, acid-etched disks, and least in plain titanium disks. This comparison is significant with a mean of 69.247 (*p* < 0.001), (Table 3 and Figure 6). On comparison of roughness between the groups, the mean values of chitosan-coated disks were the highest. This comparison was significant with a mean of 59.375 (*p* < 0.001) (Table 4 and Figure 4). Surface characteristics of the modified titanium surfaces before fibroblast cell seeding were done using a scanning electron microscope with 20 *μ*m surface area with 1000x magnification (Figure 8) and after 120 hr (day 5) of fibroblast seeding at 1 *μ*m (Figure 9). Protein assessment was done on day 1 and day 5 for all the titanium disks (Table 5). Paired *t*-test was done to compare the protein levels on day 1 and day 5. Decreased protein concentration was seen on day 5. On comparison of the mean values of protein concentration for days 1 and 5, the mean values of day 1 were higher with a difference of 0.2483612 which is statistically significant with *p* < 0.001 (Table 6).

## 4. Discussion

According to Tobias P. Kunzler [6], osteoblasts and human fibroblasts exposed to a range of roughened surfaces, osteoblasts chose the rougher part, whereas fibroblasts favored the smoother part of the roughness gradient. In this sense, roughness gradients served as the important control factor for cell response to surface roughness. According to L. Ponsonnet [7], nickel titanium surfaces have similar roughness to commercially pure titanium and titanium vanadium alloy surfaces. So, in a nickel titanium surface that is smoother, there will be a higher proliferation rate for fibroblasts. The present study showed results that are similar to those of Tobias and Ponsonnet, where fibroblasts showed decreased attachment with increasing roughness. A comparison of roughness between the five groups showed that the chitosan-coated titanium surface had the highest roughness, followed by the acid-etched surface, sandblast surface, polydopamine surface, and least for the plain surface. In a previous study done by Martin et al. [8], the chitosan films remained attached when stressed in the ultrahigh vacuum required for X-ray photoelectron spectroscopy. This method showed more tightly bound chitosan to the titanium surface. Their study included coating of the chitosan films on two differently surface treated metals which did not affect the chitosan coating. According to Chua et al. [9], surface treatment of pure titanium was done by coating it first with dopamine followed by glutaraldehyde followed by the chitosan coating. In the present study, the same method of coating the titanium disks with chitosan was followed after which the contact angle and surface roughness were evaluated. In a study done by Kononen et al. [10], the number of fibroblasts after 7 days showed an increase of almost three times on polished surfaces as compared to blasted titanium surfaces. The investigations of Cochran et al. [11] revealed a decrease in the number of fibroblasts by a factor of 1.4 after 7 days and by 2 after 9 days for cells cultured on blasted and etched titanium disks. However, in the present study, a decrease in the fibroblast growth was seen within 5 days. Bradford's protein assay was conducted, and protein assessments were done at two time points: day 1 and day 5. In comparison, the protein concentration on day 1 was higher than the protein concentration on day 5. This indicated that the decrease in the protein concentration on day 5 was associated with the cell death that occurred in all the groups of samples. A longer duration of this study will help us evaluate the cell growth curve. When the decrease in protein concentration on the 5th day was compared in different groups, it was found to be the least in acid-etched surface followed by sandblasted surface which indicates that there is more cell death in these two groups. Chitosan-coated surface showed almost similar decreases in the protein concentration but was slightly better than acid-etched and sandblasted disks. The protein concentration showed the lowest value in polydopamine-coated titanium at day 5 which meant that there was least cell death and more cell viability in polydopamine-coated titanium.

Among the surfaces, the least protein concentration is seen on the polydopamine-coated surface on both the 1st day and 5th day, which shows decreased cell death and increased viability. Hence, emphasis can be laid on the fact that the protein concentration on the first day (cell attachment) and the protein concentration on the 5th day (cell death) were on polydopamine-coated surfaces which may be attributed to its cell adhesion properties. Fibroblasts from human gingival tissue were cultured in vitro to get a close simulation of an in vivo condition. In this study, material type as well as surface processing techniques can have an impact on gingival cell adhesion strength. Moreover, as this study was performed in an artificial environment, the findings should be verified further by in vivo research for better substantiation of the results obtained. The osteoblast attachment to the threads of the orthodontic microimplant should be minimal or nil as there is no osseointegration warranted in orthodontics implants which is not the case with in vivo implants.

## 5. Conclusion

Surface modification of titanium can help to improve the surface and biological properties of titanium alloys. The roughness gradient used as a control to compare the attachment of the fibroblasts showed that polydopamine coatings on titanium disks are the most favorable surface modification for orthodontic implants followed by plain titanium. The rougher surface showed more affinity for the osteoblasts, whereas the smoother surfaces showed more fibroblast adhesion. In clinical instances requiring more osseointegration, chitosan coatings can be suggested to be effective. Titanium exposed to a variety of surface modifications and alterations, when seeded with fibroblasts, showed increased attachment of cells to the smoother surfaces of the titanium disks as compared to the rougher surfaces. Based on the protein concentration levels on days 1 and 5, it can be concluded that polydopamine-coated surfaces are the most favorable for cell viability, whereas a chitosan–polydopamine coating, acid etching, or sandblasting should be considered as the most preferred choices of surface modification for increased osseointegration.

## Figures and Tables

**Table 1:** One-way ANOVA with post hoc Tukey test for comparison of contact angle.

Variable	Comparison of	Comparison with	Mean difference	Std. error	*P* value
Contact angle	Group A	Group B	−16.17	3.4	0.0010
		Group C	−55.79	3.4	<0.001
		Group D	−23.49	3.4	<0.001
		Group E	−27.97	3.4	<0.001
	Group B	Group C	−39.62	3.4	<0.001
		Group D	−7.32	3.4	0.2530
		Group E	−11.8	3.4	0.021
	Group C	Group D	32.3	3.4	<0.001
		Group E	27.2	3.4	<0.001
	Group D	Group E	−4.47	3.4	0.699

**Table 2:** One-way ANOVA with post hoc Tukey test for comparison of roughness.

Variable	Comparison of	Comparison with	Mean difference	Std. error	*P* value
Roughness	Group A	Group B	−124.6	4.4	<0.001
		Group C	−75.06	4.4	<0.001
		Group D	−155.6	4.4	<0.001
		Group E	−18.5	4.4	0.004
	Group B	Group C	49.53	4.4	<0.001
		Group D	−30.42	4.4	<0.001
		Group E	106.08	4.4	<0.001
	Group C	Group D	−79.95	4.4	<0.001
		Group E	56.54	4.4	<0.001
	Group D	Group E	136.5	4.4	<0.001

**Table 3:** Data of contact angle of five groups of samples (*n* = 5) (mean, SD, and *P* values of contact angle).

Groups	*N*	Mean	SD	Statistic (*F*)	*P* value
Group A	5	24.816	5.69	69.247	<0.001
Group B	5	40.988	5.77		
Group C	5	80.612	6.23		
Group D	5	48.312	5.76		
Group E	5	52.79	3.48		
Total	25	49.5036	19.26		

**Table 4:** Data of roughness assessment of titanium alloy samples of five groups (mean, SD, and *P* values of roughness).

Groups	*N*	Mean	SD	Statistic (*F*)	*P* value
Group A	5	39.82	5.6419855	448.393	<0.001
Group B	5	164.42	5.7846348		
Group C	5	114.886	10.1315586		
Group D	5	194.84	7.5899934		
Group E	5	58.34	4.5665085		
Total	25	114.4612	61.0239609		

**Table 5:** Data of protein concentrations on day 1 and day 5 in all five groups.

Groups	Protein concentration (mg/mL)	
	Day 1	Day 5
1	0,86	0.49
2	0.74	0.67
3	0.73	0.57
4	0.76	0.48
5	0.60	0.25

**Table 6:** Paired *t* test for protein levels.

Protein concentration	*N*	Mean	SD	Paired differences		*t*	*df*	*P* value
				Mean difference	SD			
Day 1 protein concentration	5	0.739951	0.092896	0.248361	0.129392	4.292	4	**0.013**
Day 5 protein concentration	5	0.491589	0.154645					

## Data Availability

Data will be made available at journals request.
